# Post-streptococcal glomerulonephritis leading to posterior reversible encephalopathy syndrome: a case report

**DOI:** 10.1186/1756-0500-7-644

**Published:** 2014-09-13

**Authors:** Madura Adikari, Dilani Priyangika, Indika Marasingha, Sharmila Thamotheram, Gayani Premawansa

**Affiliations:** Colombo North Teaching Hospital, Ragama, Sri Lanka

**Keywords:** Posterior reversible encephalopathy syndrome, T2 weighted magnetic resonance imaging, Post-streptococcal glomerulonephritis, Hypertensive encephalopathy, Sri Lanka

## Abstract

**Background:**

Posterior reversible encephalopathy syndrome is a clinical radiographic syndrome of heterogeneous etiologies. Developing hypertensive encephalopathy following post-streptococcal glomerulonephritis is a known but uncommon manifestation and developing posterior reversible encephalopathy syndrome in such a situation is very rare. We report a case with contrast-enhanced computed tomography and magnetic resonance imaging findings of posterior reversible encephalopathy syndrome in the background of acute post-streptococcal glomerulonephritis.

**Case presentation:**

A thirteen- year -old Sri Lankan boy presented with a focal fit by way of secondary generalization with duration of 10 minutes, and developed 2 similar fits subsequently following admission. He later developed severe hypertension with evidence of glomerulonephritis, which was diagnosed as acute post-streptococcal glomerulonephritis. A contrast-enhanced computed tomography imaging of brain done on day-3 revealed non-enhancing low-attenuating areas in fronto -parietal regions. A T2 weighted film of magnetic resonance imaging was done on day-10 of the admission and found to have linier sub-cortical hyper intensities in both parietal regions which were compatible with the radiological diagnosis of posterior reversible encephalopathy syndrome.

**Conclusion:**

Post-streptococcal glomerulonephritis is an important cause of acute nephritic syndrome especially in children. This case report illustrates a rare association of posterior reversible encephalopathy syndrome in a patient with post-streptococcal glomerulonephritis.

## Background

Posterior reversible encephalopathy syndrome (PRES) is a clinical radiographic syndrome of heterogeneous etiologies which is increasingly recognized and reported in case reports and case series. Several medical conditions are reported to be associated with PRES and some of these include hypertensive encephalopathy, acute or chronic renal diseases, thrombotic thrombocytopenic purpura, hemolytic and uremic syndrome, eclampsia, vasculitis and porphyrias. Developing hypertensive encephalopathy following post-streptococcal glomerulonephritis (PSGN) is a known but uncommon manifestation in children [1], and developing PRES in such a situation is very rare [2]. Here we would like to report such a case in a 13-year-old boy who was admitted to our medical unit.

### Case presentation

A 13-year-old Sri Lankan boy presented to our medical unit with a sudden onset tonic-clonic convulsion of the right arm with secondary generalization resulting in loss of consciousness lasting about 10 minutes. He developed two similar focal fits after admission of which the durations were about 5 minutes each. When further questioning from the parents, it was revealed that he had developed sore throat and fever 2 weeks prior to admission and were resolved with treatment including an oral antibiotic. He was fever free for about one week but he complained of recurrent frontal headaches. He had not noticed any gross hematuria, dysuria or decreased urine output prior to admission. On the day of admission the patient had developed repeated episodes of vomiting and, increased sleepiness. He also noticed to have transient visual disturbances which he explained as distorted sizes of the images. The patient also gave a history of an infected wound over right ankle that he had 2 weeks back, which was healed by the time of admission.

On admission he was drowsy but rousable with a Glasgow coma scale (GCS) of 13/15. He had mild periorbital swelling, without clinically detectable dependant edema. His throat was not inflamed and his thyroid gland was normal. He didn’t have any signs of meningeal irritation or any focal neurological signs. His blood pressure was 130/80 mmHg on admission with a significant protein urea detected by dipstick. Within 2 hours his blood pressure raised up to 200/100 mmHg and he was admitted to the intensive care unit (ICU) for close monitoring and further management. While in the ICU intravenous antihypertensive drugs were used to control the blood pressure. Later blood pressure was controlled with oral antihypertensive medications. His urinalysis reported as moderately field full red cells with dysmorphic red cells of 15% suggestive of hematuria of glomerular origin and occasional pus cells but no cellular casts. His anti streptolysin O titre (ASOT) reported as more than 200 U/mL and was considered significant, urine and the throat swab cultures were sterile. He had normal serum creatinine and electrolytes; however his blood urea was elevated up to 26.7 mmol/L despite adequate hydration. His serum protein was low. Diagnosis of acute PSGN was made and he was started on intra venous (IV) penicillin.

During the second day of ICU his urine output started declining to less than 0.5 mL/kg/h. His peri orbital swelling worsened and developed bilateral plural effusions and ascites. He was started on IV furosemide and strict fluid balance was maintained. His blood urea started to rise despite adequate hydration but other renal functions including serum creatinine remained normal. He underwent ultrasound scan of the abdomen on day-2 of the admission and showed ultrasonic evidence of acute renal parenchymal disease, ascites and bilateral plural effusions. Blood picture was suggestive of acute bacterial infection but the blood cultures and throat swab cultures were sterile. He underwent 24 hour urine collection and found to have sub-nephrotic range protein urea of 0.6 g/24 h. An urgent electro encephalogram (EEG) was done on admission and showed changes suggestive of encephalopathy secondary to recurrent fits, he was started on sodium valproate and phenytoin sodium by the neurologist.

**Figure 1:**
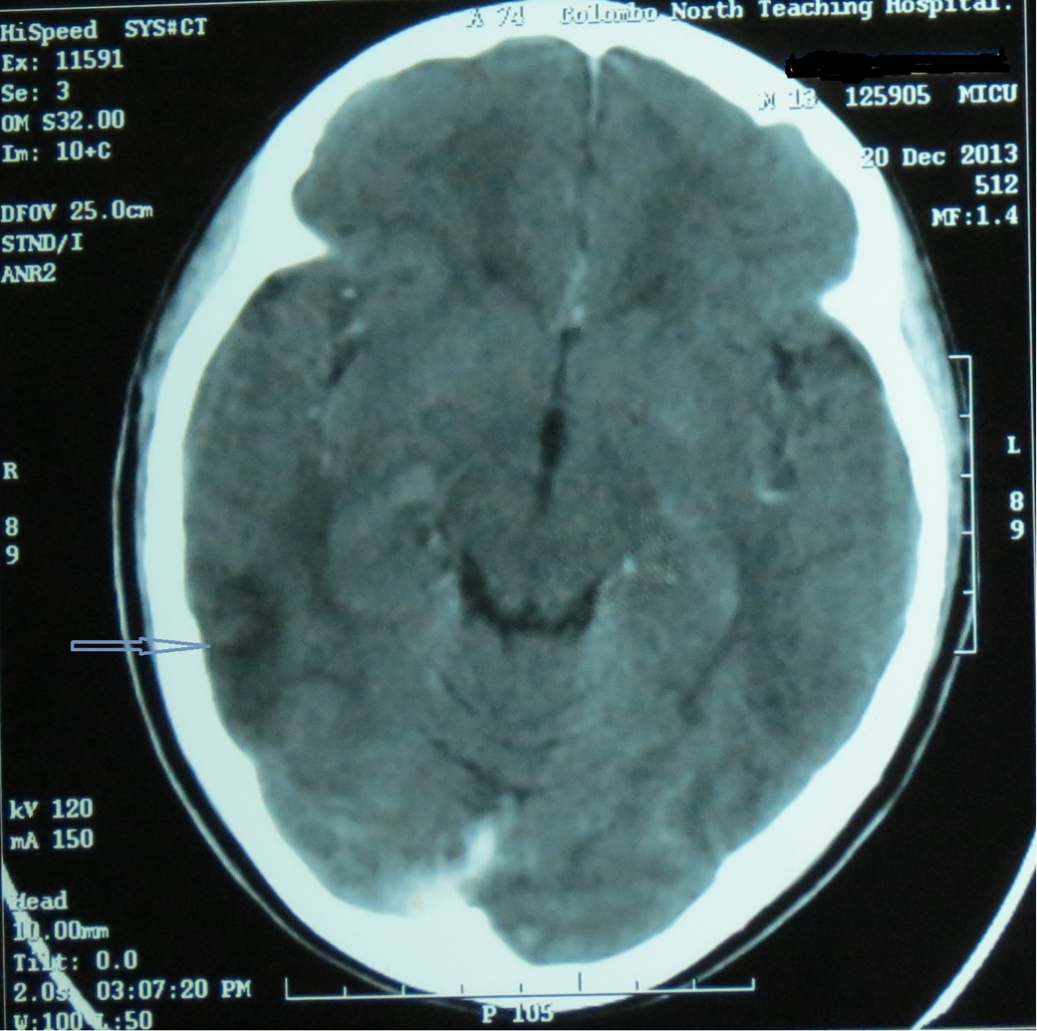
**A contrast-enhanced computed tomography scan of brain done on day-3 showed non enhancing low attenuating areas in fronto -parietal regions (arrow).**

**Figure 2:**
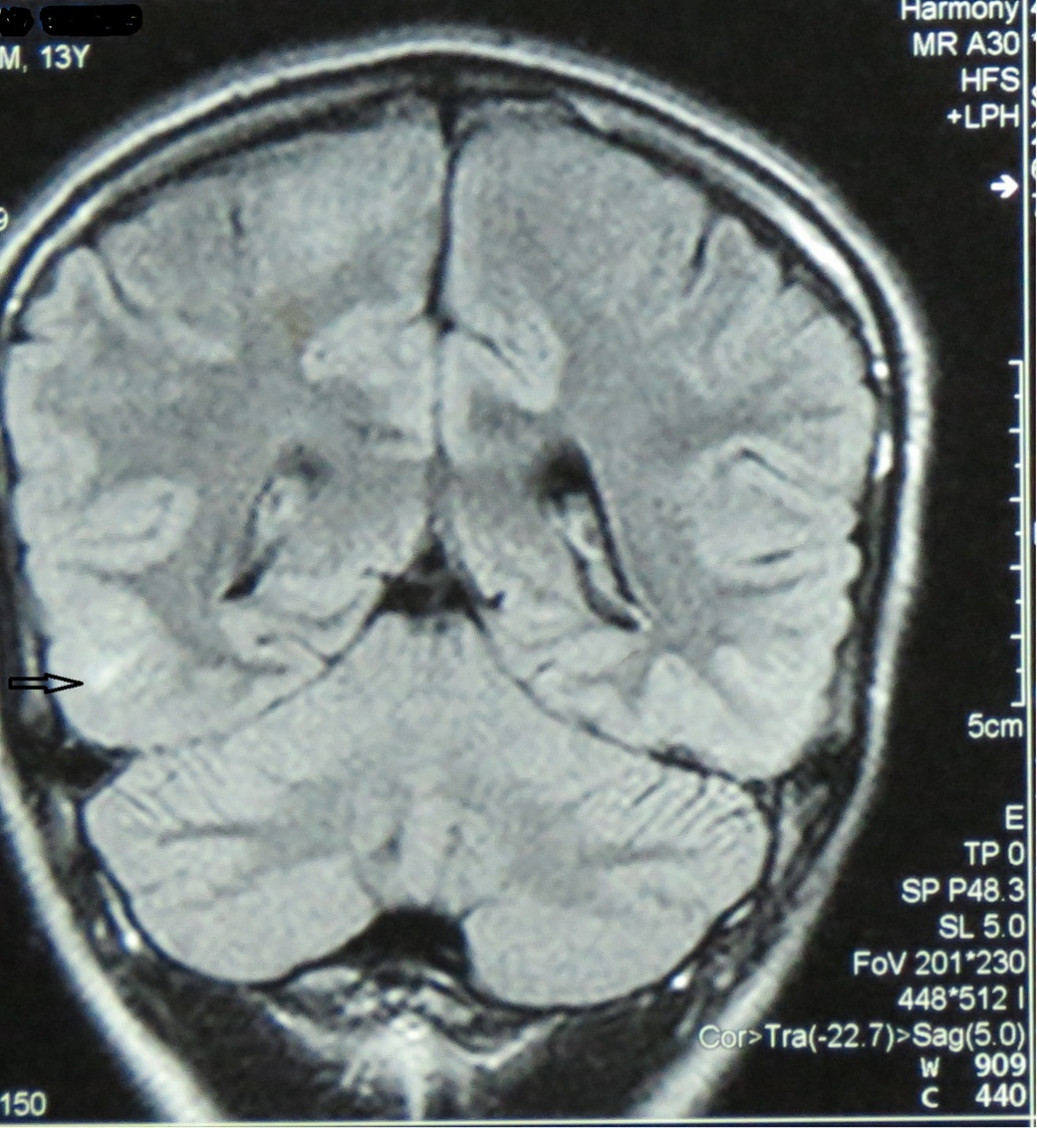
**Magnetic resonance imaging scan done day-10 of the admission showed linier sub-cortical hyper intensities (arrow) in both parietal regions.**

A contrast-enhanced computed tomography (CT) of brain done on day-3 and revealed non-enhancing low-attenuating areas in fronto -parietal regions (Figure 1). There was no abnormal meningeal enhancement or focal lesions. A T2 weighted image of magnetic resonance imaging (MRI) was taken on day-10 of the admission and found to have linier sub-cortical hyper intensities in both parietal regions which was consistent with the radiological diagnosis of PRES. MRI findings were less intensive than CT finding suggesting recovery of the cerebral insults during that period (Figure 2). As cerebral vascuitis was also a possibility, erythrocyte sedimentation rate (ESR), anti nuclear antibody (ANA) and anti neutrophil cytoplasmic antibody (ANCA) were done and found to be negative. After 5 days of ICU stay followed by 2 days of inward stay, patient's blood pressure was well controlled with oral captopril. He had a satisfactory urine output without diuretics and no clinical or biochemical evidence of acute kidney injury. His hematuria was settling and he was seizure free. His vision had returned to normal. He was discharged with the antihypertensive and anti-epileptics and planned to be reviewed weekly in the outpatient clinic. After 3 weeks, his hematuria had totally resolved according to repeat urinalysis and antihypertensive drug was gradually tailed off whilst patient was normotensive. He underwent a repeat MRI scan 3 weeks later and the above radiological manifestations disappeared, thus it was reported as normal.

## Discussion

The possible diagnosis of PRES following hypertensive encephalopathy secondary to acute PSGN was arrived based on clinical presentation, laboratory and imaging data. The early clinical presentation of PRES such as headaches, altered mental status, seizures, and visual loss thought to be associated with white matter vasogenic edema predominantly affecting the posterior occipital and parietal lobes of the brain [3]. PRES has several etiologies, hypertensive encephalopathy, acute or chronic renal diseases, thrombotic thrombocytopenic purpura, hemolytic and uremic syndrome, eclampsia, vasculitis and porphyria being the most commonly reported associated diseases. Several drugs have been reported to cause PRES and ciclosporin after organ transplantation is a well known cause [4].

CT and MRI imaging is the mainstay in the diagnosis of PRES. The parietal and occipital lobes are most commonly affected, followed by the frontal lobes, the inferior temporal-occipital junction, and the cerebellum [5, 6]. As in this case initial contrast CT imaging may appear like infarctions. In such cases with the clinical suspicion, further imaging of MRI with T1, T2 and diffusion-weighted imaging (DWI) will be helpful in the diagnosis. On MRI brain, bilateral symmetrical edema in the parieto-occipital region is hyper-intense on T2-weighted and hypo-intense on T1-weighted sequences [7]. DWI can differentiate this condition from other major diseases such as infarction that are diffusion restricted [8]. These changers are usually reversible over time.

The pathophysiology of PRES is unclear and under debate, but it is thought to be related to disordered cerebral auto regulation which leads to fluid leakage into the brain leading to cerebral edema [9]. Hypertensive encephalopathy is one of the most common causes of PRES, but acute PSGN leading to hypertensive encephalopathy is a rare manifestation. Although seizures occurring with severe hypertension could be due to hypertensive encephalopathy, seizures are one of the commonest manifestations of PRES. In the majority of cases seizures are generalized but as in this case it could also be a focal onset with secondary generalization and may repeatedly occur where some patients may progress to status epilepticus [10].

## Conclusion

Acute PSGN is a well known sequel of streptococcal infections and one of the commonest causes of acute nephritic syndrome especially in children, which could be complicated with hypertensive emergencies [11]. Clinical radiographic syndrome of PRES is not a well described presentation in acute PSGN. Above case illustrates a rare occurrence of PRES in the background of acute PSGN in a 13-year-old child.

### Consent

Written informed consent was obtained from the patient's father for publication of this case report and any accompanying images. A copy of the written consent is available for review by the Editor-in-Chief of this journal.

## Abbreviations

ANA: Anti nuclear antibody
ANCA: Anti -neutrophil cytoplasmic antibody
CT: Computed tomography
DWI: Diffusion weighted images
EEG: Electro encephalography
ESR: Erythrocyte sedimentation rate
GCS: Glasgow coma scale
ICU: Intensive care unit
IV: Intra venous
MRI: Magnetic resonance imaging
PRES: Posterior reversible encephalopathy syndrome
PSGN: Post-streptococcal glomerulonephritis.
